# Anandamide Reduces Intracellular Ca^2+^ Concentration through Suppression of Na^+^/Ca^2+^ Exchanger Current in Rat Cardiac Myocytes

**DOI:** 10.1371/journal.pone.0063386

**Published:** 2013-05-07

**Authors:** Qian Li, Na Cui, Yuanjie Du, Huijie Ma, Yi Zhang

**Affiliations:** 1 Department of Physiology, Hebei Medical University, Shijiazhuang, China; 2 Department of Reproduction, Second Hospital of Hebei Medical University, Shijiazhuang, China; Universität Regensburg, Germany

## Abstract

**Purpose:**

Anandamide, one of the endocannabinoids, has been reported to exhibit cardioprotective properties, particularly in its ability to limit the damage produced by ischemia reperfusion injury. However, the mechanisms underlying the effect are not well known. This study is to investigate whether anandamide alter Na^+^/Ca^2+^ exchanger and the intracellular free Ca^2+^ concentration ([Ca^2+^]_i_).

**Methods:**

Na^+^/Ca^2+^ exchanger current (I_NCX_) was recorded and analysed by using whole-cell patch-clamp technique and [Ca^2+^]_i_ was measured by loading myocytes with the fluorescent Ca^2+^ indicator Fura-2/AM.

**Results:**

We found that I_NCX_ was enhanced significantly after perfusion with simulated ischemic external solution; [Ca^2+^]_i_ was also significantly increased by simulated ischemic solution. The reversal potential of I_NCX_ was shifted to negative potentials in simulated ischemic external solution. Anandamide (1–100 nM) failed to affect I_NCX_ and [Ca^2+^]_i_ in normal solution. However, anandamide (1–100 nM) suppressed the increase in I_NCX_ in simulated ischemic external solution concentration-dependently and normalized I_NCX_ reversal potential. Furthermore, anandamide (100 nM) significantly attenuated the increase in [Ca^2+^]_i_ in simulated ischemic solution. Blocking CB1 receptors with the specific antagonist AM251 (500 nM) failed to affect the effects of anandamide on I_NCX_ and [Ca^2+^]_i_ in simulated ischemic solution. CB2 receptor antagonist AM630 (100 nM) eliminated the effects of anandamide on I_NCX_ and [Ca^2+^]_i_ in simulated ischemic solution, and CB2 receptor agonist JWH133 (100 nM) simulated the effects of anandamide that suppressed the increase in I_NCX_ and [Ca^2+^]_i_ in simulated ischemic solution. In addition, pretreatment with the Gi/o-specific inhibitor pertussis toxin (PTX, 500 ng/ml) eliminated the effects of anandamide and JWH133 on I_NCX_ in simulated ischemic solution.

**Conclusions:**

Collectively, these findings suggest that anandamide suppresses calcium overload through inhibition of I_NCX_ during perfusion with simulated ischemic solution; the effects may be mediated by CB2 receptor via PTX-sensitive Gi/o proteins. This mechanism is importantly involved in the anti-ischemia injury caused by endocannabinoids.

## Introduction

Anandamide is one of endocannabinoids, which are involved in the regulation of neurobehavioral, gastrointestinal, stress and anxiety, and cardiovascular functions physiological and pathological stages [1]–[4]. Up to date, at least two types of cannabinoid (CB) receptors, CB1 and CB2 receptor, have been identified and are widely expressed in many tissues including cardiac myocytes [5], [6]. Both receptor types belong to a group of seven transmembrane-spanning receptors and are coupled to Gi/o protein [4]. Anandamide limits the damage during ischemia–reperfusion in rat isolated hearts through various mechanisms [7], [8]. Also, anandamide has been found to protect the heart from adrenaline-induced arrhythmias [9] and arrhythmias induced by ischemia-reperfusion [10]. In our recent study, we found that the anandamide exerts anti-arrhythmia action through inhibition of L-type Ca^2+^ currents [11] and transient outward K^+^ currents [12].

Na^+^/Ca^2+^ exchanger (NCX) is expressed in almost all the tissues including cardiac myocytes [13]. In addition to being found in the plasma membranes, NCX is expressed in the mitochondria and endoplasmic reticulum of excitable cells [14], [15].The NCX is an membrane protein that export Ca^2+^ from cells and import Na^+^ into the cells. The NCX removes a Ca^2+^ ion in exchange for the import of three Na^+^ ions and is considered one of the most important mechanisms for Ca^2+^ removal in cardiac muscle [16], [17]. Activation of NCX at forward mode (Ca^2+^ extrusion) produces an inward current (1 Ca^2+^ extrusion, 3 Na^+^ influx) and an outward currents is induced (3 Na^+^ extrusion, 1 Ca^2+^ influx) if NCX works at the reverse mode (Ca^2+^ influx) [13], [18]. The NCX may operate in both forward and reverse directions simultaneously in different areas of the cell, depending on the combined effects of Na^+^ and Ca^2+^ gradients [17]. It has been shown that the rise in cytosolic free Ca^2+^ concentration [Ca^2+^]_i_ during ischemia is due to Ca^2+^ entry by reverse-mode NCX [18]. It seems that NCX plays a central role in controlling Ca^2+^ homeostasis in cardiomyocytes, especially during cardiac ischemia.

Anandamide can protect heart against ischemia-reperfusion injury. However, it is not clear if NCX is involved an anandamide-induced protective effect during ischemia-reperfusion injury to alleviate Ca^2+^ overload? Therefore, in this study, by using whole-cell patch clamp and Fura-2/AM fluorescence ratio image method, we explored the effects of anandamide on I_NCX_ and [Ca^2+^]_i_ in ventricular myocytes.

## Materials and Methods

### Animals

We carried out this study in adult male Sprague-Dawley rats (weighting 230–280 g) obtained from the Experimental Animal Center of Hebei Province. This study was performed conforming to the Guide for the Care and Use of Laboratory Animals described by Directive 2010/63/EU of the European Parliament. Animal work was approved by the Ethics Committee for Animal Experiments of the Hebei Medical University, in compliance with NIH Guidelines, and carried out in compliance with China Government Guidelines.

### Isolation of ventricular myocytes

Single ventricular myocytes were isolated from the heart of adult rat by using enzymatic dissociation as described previously [11]. Briefly, the rats were anesthetized by intraperitoneal injection of sodium pentobarbital (150 mg/kg) and heparin (300 U/kg). The depth of anesthesia was confirmed by the lack of motor reflexes during noxious pinch (blunt forceps) of the hindpaw, the forepaw, and the ear. Body temperature was maintained at 37°C. The rat heart was excised and retrogradely perfused on a Langendorff apparatus with oxygenated ice-cold Ca^2+^-free Tyrode's solution via the aorta at a perfusion rate of 4 ml/min for 5 min. The Ca^2+^-free Tyrode's solution contained (in mM) NaCl 135, KCl 5.4, MgCl_2_ 1.0, NaH_2_PO_4_ 0.33, glucose 5, and HEPES 10 (pH was adjusted to 7.4 with NaOH). Then, the heart was perfused with Tyrode's solution containing CaCl_2_ (34 µM) and collagenase II (300 mg/L) at 37 °C for 12 min. Finally, the left ventricle was removed and teased into smaller pieces in Kreb's solution containing (in mM) KOH 80, KCl 40, NaH_2_PO_4_ 25, MgSO_4_ 3, glutamic acid 50, taurine 20, EGTA 1, HEPES 10, and glucose 10 (pH was adjusted to 7.4 with KOH). Single myocytes were harvested after filtration through a nylon mesh (pore size 200 µm) and stored in Kreb's solution at room temperature for at least 1 h, then the concentration of Ca^2+^ in Kreb's solution was gradually increased to 1.0 mM before the experiment.

### Electrophysiological measurements

**Figure 1:**
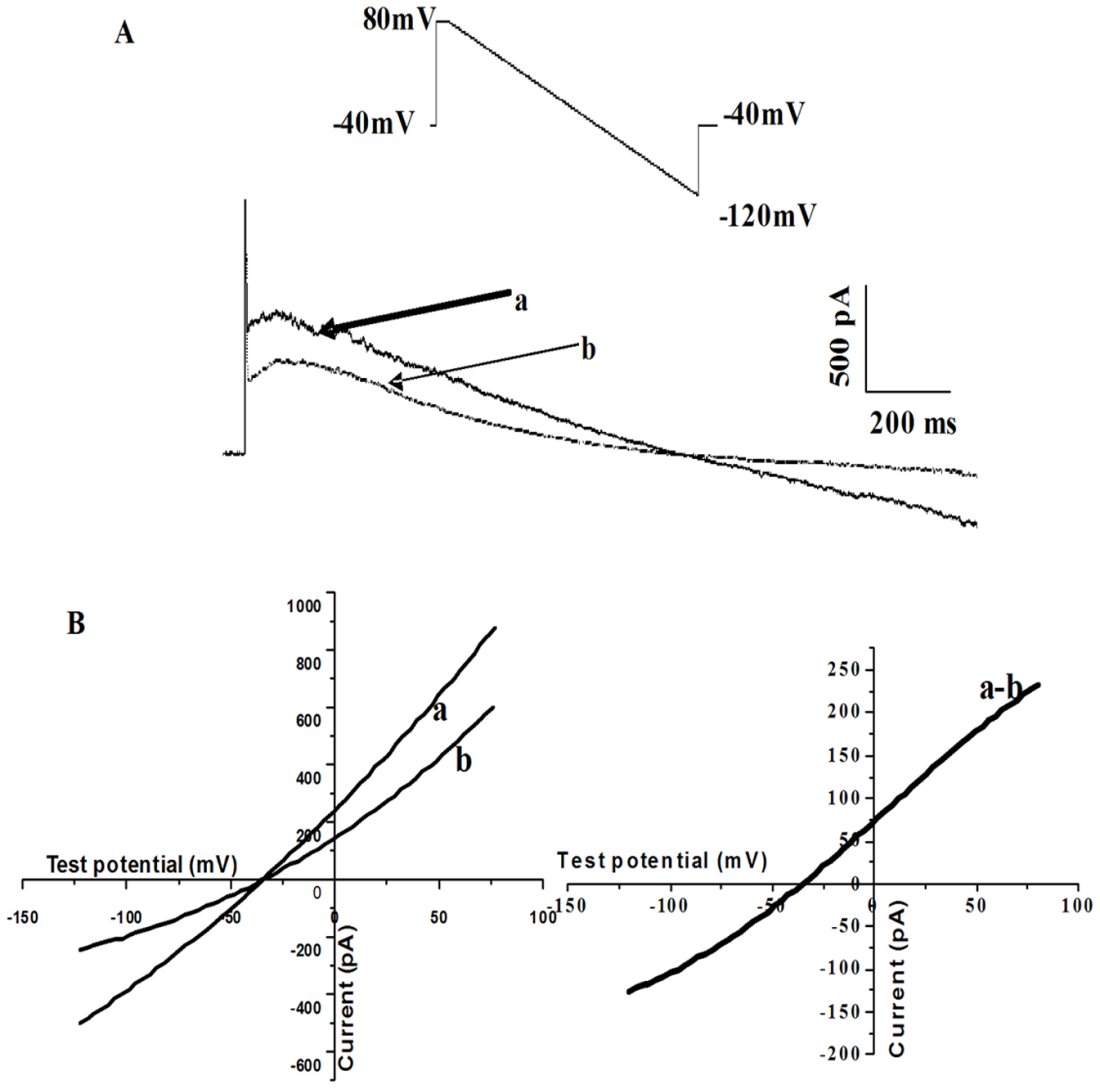
Recording of Na^+^/Ca^2+^ exchanger current (I_NCX_) in rat ventricular myocytes. (A) Voltage protocol used for measuring I_NCX_, the typical membrane currents obtained in a myocyte in the absence (a) or presence 5 mM Ni^2+^ (b). Currents were elicited by ramp pulse from +80 mV to −120 mV at a rate of 90 mV/s. (B) Current–voltage (I-V) relationship before (a) and after (b) application of 5 mM Ni^2+^. (C) I-V relationship for I_NCX_ (a–b).

Isolated ventricular myocytes were placed in a recording chamber mounted on the stage of an inverted microscope (Olympus, Japan). After 30 min settling to the bottom of chamber, the myocytes were superfused with the external solution for 10 min at a rate of 2–3 ml/min at room temperature. Whole-cell recordings were performed on these cells and membrane current was assessed by using Axopatch 200B patch-clamp amplifier and Digidata 1200B acquisition board (Axon Instruments, Foster City, CA, USA). Patch-pipettes were pulled by a micropipette puller (P-97, Sutter Instrument Co., Novato, CA, USA) with a resistance of 2 to 5 MΩ when it was filled with electrode internal solution containing (in mM) 65 CsCl_2_, 20 NaCl, 5 MgATP, 6 CaCl_2_, 4 MgCl_2_, 10 HEPES, 20 tetraethyl ammonium chloride (TEA), and 21 EGTA (pH 7.4 adjusted with CsOH) [19]. Only the rod-shaped cells with visible striations were selected for recording. Liquid junction potential between the pipette and external solution was corrected after the pipette tip dipped into the external solution. After forming a ‘gigaseal’, the membrane was ruptured by a gentle suction to obtain the whole-cell configuration. Membrane currents were recorded by using a standard voltage-ramp protocol. From a holding potential of −40 mV, a 2-s voltage ramp (from −120 mV to +80 mV at 90 mV/s) was used to elicit I_NCX_. Ni^2+^ (5 mM) was added to block the I_NCX_. The protocol was repeated in the presence of 5 mM Ni^2+^ to obtain Ni-insensitive currents. I_NCX_ was measured as the Ni-sensitive currents which were calculated as a subtraction of residual currents in the presence of Ni^2+^ from the total currents (Figure.1). In the whole-cell patch-clamp recording, L-type Ca^2+^, K^+^, and Na^+^–K^+^ pump currents were blocked by specific blockers: nifedipine for L-type Ca^2+^, CsCl for K^+^, and ouabain for Na^+^–K^+^ pump currents, respectively.

I_NCX_ was recorded when myocytes were superfused by normal external solution and simulated ischemic external solution to mimic ischemic environment [19]–[21]. The normal external solution for I_NCX_ recording contained (in mM) NaCl 140, CaCl_2_ 2.0, MgCl_2_ 2.0, HEPES 5.0, glucose 10, Oubain 0.02, BaCl_2_ 1.0, CsCl_2_ 2.0, nifedipine 0.001 (pH was adjusted with NaOH to 7.4; gassed with 100% O_2_). Simulated ischemic external solution contained (in mM): NaCl 137.8, MgCl_2_ 1.0, CaCl_2_ 1.8, NaHCO_3_ 3.8, NaH_2_PO_4_ 0.9, Oubain 0.02, BaCl_2_ 1.0, CsCl_2_ 2.0, nifedipine 0.001, Sodium lactate 20 and was gassed with 95% N_2_ and 5% CO_2_ (pH 6.8) for 30 min. Glibenclamide 20 µM was added to external solutions to block I_KATP_. Cariporide 10 µM, a selective Na^+^/H^+^ exchanger (NHE) inhibitor, was also added to external solutions to block Na^+^/H^+^ exchanger current (I_NHE_) [22].

### Measurement of [Ca^2+^]_i_

Myocytes were incubated with 2 µM Fura-2/AM for 40 min at 37°C to load Fura-2/AM into the cell. Briefly, the coverslip was placed in a small superfusion chamber on the stage of an inverted microscope (IX71, Olympus, Tokyo, Japan). [Ca^2+^]_i_ was measured with a video-imaging-system (Till Photonics, Munich, Germany). Cells were illuminated alternately at 340- and 380-nm excitation wavelengths, excitation light was provided by a monochromator (Till Photonics). Then, 500-nm emission light images were captured by an image-intensifying, charge-coupled device (CCD) camera (SensiCam, PCO, Kelheim, Germany) and digitized by an image processing system (TillVision, Till Photonics). The monochromator and CCD camera were controlled by TillVision software, which was also for image analysis. Ratios were converted to Ca^2+^ concentrations as previously described [23].

[Ca^2+^]_i_ was recorded when myocytes were superfused with normal Tyrode's solution and simulated ischemic solution to mimic ischemic environment [19]–[21]. The composition of normal Tyrode's solution (in mM) was: NaCl 136.8, KCl 5.4, MgCl_2_ 1.05, CaCl_2_ 1.80, NaHCO_3_ 1.2, glucose 11.0, and Tris 5.0 (pH 7.4 ± 0.05, gassed with 100% oxygen). The composition of simulated ischemic solution (in mM) was: NaCl 137.8, KCl 8.0, MgCl_2_ 1.0, CaCl_2_ 1.8, NaHCO_3_ 3.8, NaH_2_PO_4_ 0.9, Sodium lactate 20 (pH 6.8, gassed with 95% N_2_ and 5% CO_2_). Glibenclamide 20 µM was added to solutions to block I_KATP_. Solutions were maintained at a temperature of 37.0 ± 0.5°C throughout the experiment.

### Drugs

Anandamide, JWH133, AM251, and AM630 were purchased from Cayman Chemical Company (Ann Arbor, MI, USA). Collagenase II was purchased from Invitrogen·Gibco (Grand Island, NY, USA). Fura-2/AM, glibenclamide, cariporide, SEA0400, pertussis toxin (PTX) and other reagents were purchased from Sigma-Aldrich Co (St Louis, MO, USA). Anandamide, JWH133, AM251 and AM630 were initially dissolved in dimethylsulfoxide (DMSO) and the final concentration of DMSO during the experiment was less than 0.1%. In our pilot study, we found that DMSO (up to 0.1%) alone had no effect on the electrophysiological characteristics of myocytes.

### Data analysis

Data were expressed as mean ± SEM. For electrophysiological recording data analysis were replayed and analysed by using Pclamp10.0 (Axon Instruments). [Ca^2+^]_i_ was calculated from fluorescence emission ratios obtained from 340- and 380-nm excitation of the Ca^2+^-bound and Ca^2+^-free forms of fura- according to the following formula: [Ca^2+^]  =  Kd·[(R- Rmin)/(Rmax- R)]·S, where Kd (224 nM) is the dissociation constant for the association of the deesterified Fura-2 molecule with ionized calcium; R, Rmax, and Rmin are, respectively, the fluorescence ratio (340∶380) for the sample at time t, following the addition of 100 µl of 0.1% Triton X-100 and following the addition of 100 µl of 0.5 mM EGTA to the cell suspension; S is the ratio of fluorescence at 380 nm in EGTA-treated samples over Triton X-100-treated samples. Excitation of Fura-2 and electrical stimulation were restricted to brief recording periods. Calibrations in single cells were carried out at the end of each experiment. Although the calculated values of [Ca^2+^]_i_ may be sensitive to calibration reference and other factors, the overall conclusions drawn from the data are not altered because each cell serves as its own control. The differences between groups were compared using one-way ANOVA or within groups before and after drug application using ANOVA with repeated measures. When significant differences were found using ANOVA, the *Dunnet's post hoc* test was used to determine which groups were significantly different. Statistical significance was accepted at *P*<0.05.

## Results

### Anandamide inhibited I_NCX_ in the simulated ischemic external solution but not in normal external solution

**Figure 2:**
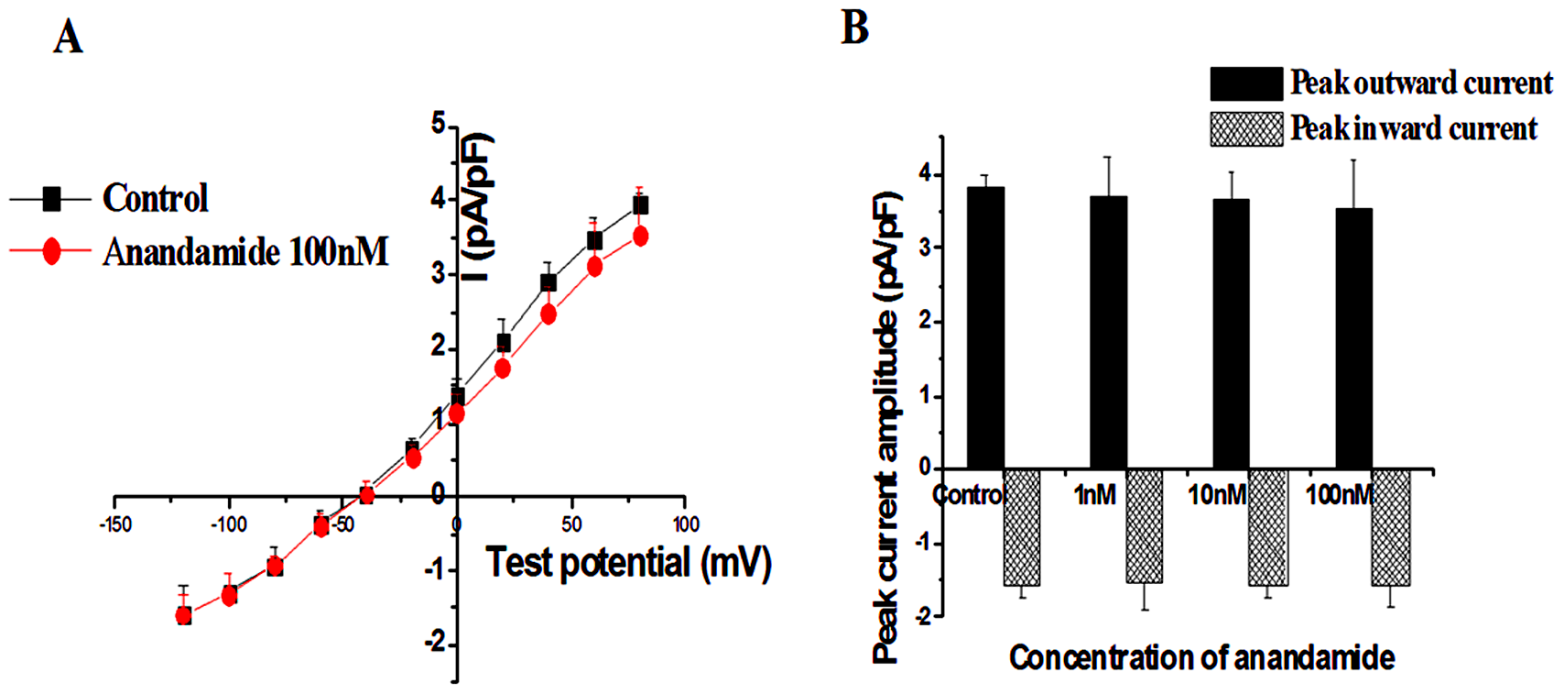
Effect of anandamide on I_NCX_ in rat ventricular myocytes in the normal external solution. (A) I-V curve of I_NCX_ in the absence and presence of 100 nM anandamide. (B) Summay data of peak outward (at +80 mV) and inward (at −120 mV) I_NCX_ from myocytes without or with 1 nM, 10 nM and 100 nM anandamide, n = 10 in each group.

I_NCX_ was recorded before and after bath application of anandamide at time points of 1, 5, 10, 15, 20, 25 and 30 min when myocytes were superfused with the normal pH (pH 7.4 ± 0.05) external solution. The current-voltage (I-V) curve of I_NCX_ had no change with application of 1 nM, 10 nM and 100 nM anandamide at 30 min (Figure. 2).

**Figure 3:**
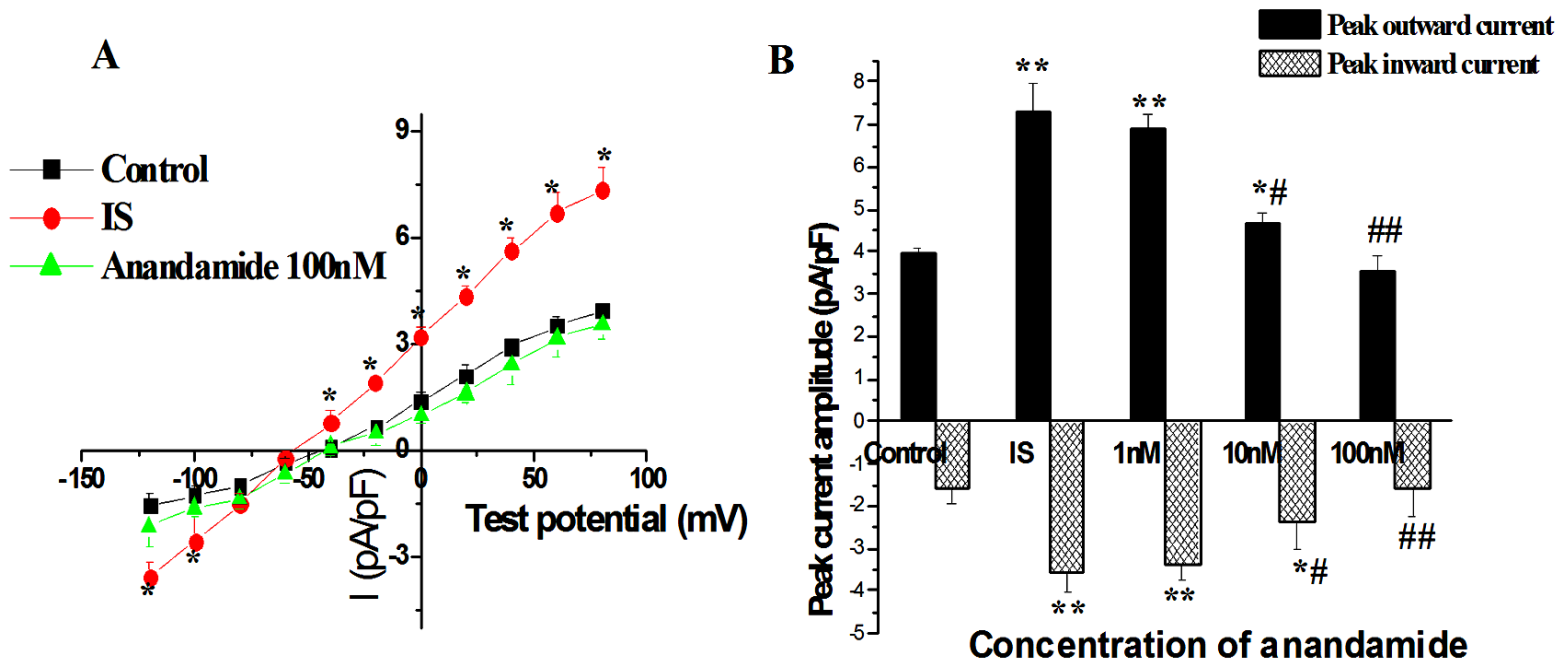
Effect of anandamide on I_NCX_ in rat ventricular myocytes in the simulated ischemic external solution. (A) I-V curve of I_NCX_ in the normal external solution (Control), in the simulated ischemic external solution (IS) and application 100 nM anandamide to the simulated ischemic external solution (Anandamide 100 nM). (B) Anandamide reduced peak outward and inward I_NCX_ in a concentration-dependent manner in the simulated ischemic external solution. * *P*<0.05 ** *P*<0.01 vs Control, **^#^** *P*<0.05 **^##^** *P*<0.01 vs IS, n = 10 in each group.

After being equilibrated by normal external solution for more than 5 min, cells were perfused with the simulated ischemic external solution (pH 6.8) for 30 min. Compared with the control currents, the current amplitude of I_NCX_ was enhanced significantly after 30 min perfusion with simulated ischemic external solution (from −1.57±0.36 to −3.58±0.45 pA/pF at −120 mV; from 3.95±0.16 to 7.31±0.66 pA/pF at +80 mV, n = 10 Figure. 3A). The reversal potential was shifted to negative membrane potentials (from −42.6±3.8 mV to −61.8±4.6 mV, *P*<0.05).

Then, we determined the effect of anandamide on the I_NCX_ in the simulated ischemic external solution. Anandamide was added to the simulated ischemic external solution 5 min before recording. The maximal effects of anandamide were measured within 8 min of initial exposure. Anandamide at concentrations of 1, 10, and 100 nM dose-dependently inhibited the outward peak currents that were decreased by 5.75 ± 3.6%, 36.3 ± 6.5%, and 52.6 ± 8.3% respectively. Furthermore, the inward peak currents were also inhibited by 6.18 ± 3.8%, 34.1 ± 5.4%, and 55.2 ± 7.9% (Figure. 3B). Application of 100 nM anandamide to the simulated ischemic external solution for 10 min, both outward and inward I_NCX_ were reduced (from −3.58±0.45 to −2.16±0.61 pA/pF at −120 mV; from 7.31±0.66 to 3.53±0.38 pA/pF at +80 mV, n = 10). In addition, the reversal potential was returned to the normal level (Figure. 3A).

### Role of CB1 and CB2 receptors in the effect of anandamide on I_NCX_ in the simulated ischemic external solution

**Figure 4:**
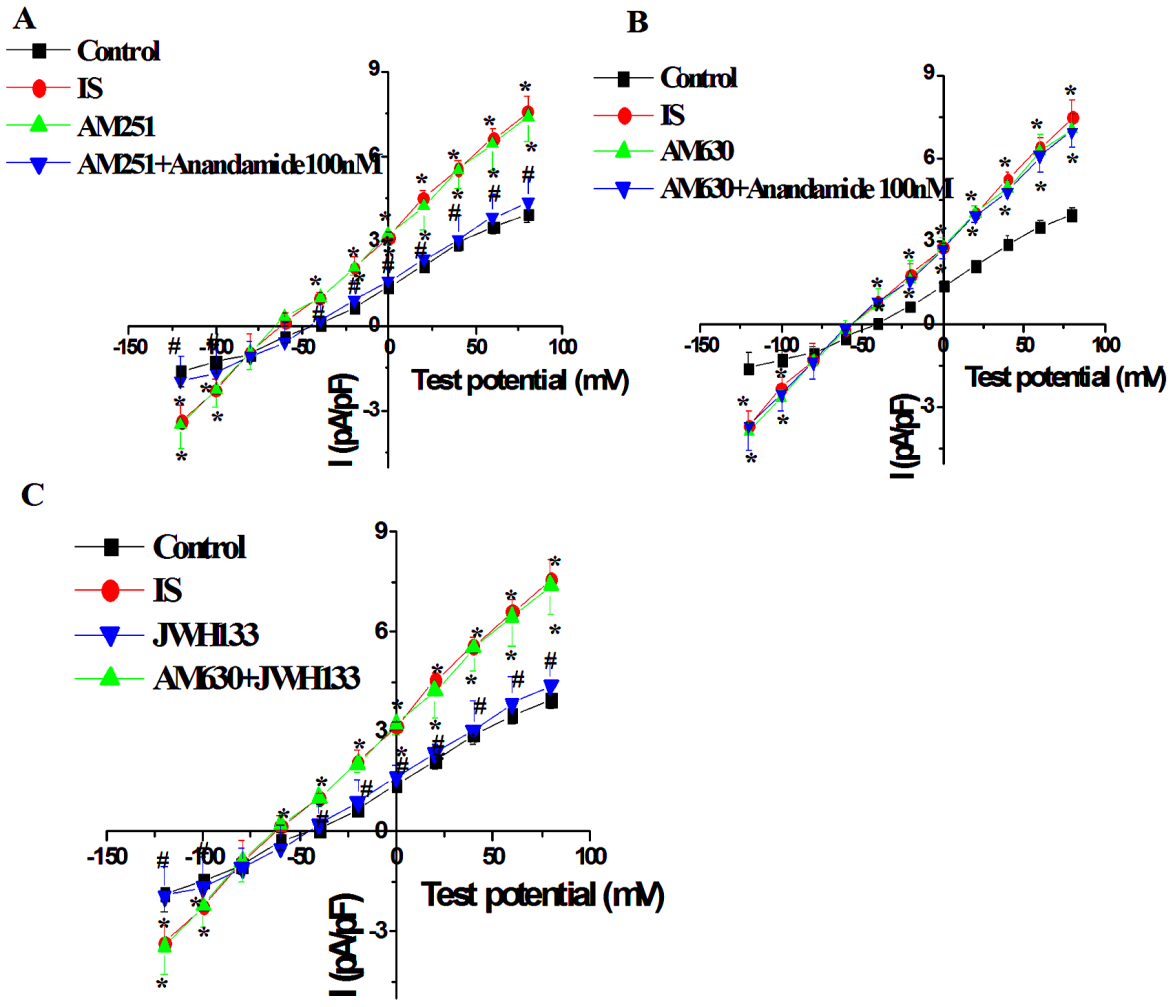
Effects of CB1 receptor antagonist AM251 (500 nM) (A) and CB2 receptor antagonist AM630 (100 nM) (B) on anandamide-induced I_NCX_ change, and CB2 receptor agonist JWH133 (100 nM) on I_NCX_ (C) in isolated rat ventricular myocytes in the simulated ischemic external solution. * *P*<0.05 vs Control, **^#^** *P*<0.05 vs IS, n = 10 in each group.

To determine the receptor subtypes that mediated the effect of anandamide on I_NCX_, the myocytes were then exposed to the simulated ischemic external solution after being perfused by normal external solution for 15 min. The effect of 100 nM anandamide on I_NCX_ in ventricular myocytes during simulated ischemia was tested after treatment with CB1 antagonist AM251 (500 nM) or CB2 antagonist AM630 (100 nM). CB1 receptor antagonist AM251 (500 nM) or CB2 receptor antagonist AM630 (100 nM) had little effect on basal I_NCX_ of cardiac myocytes in simulated ischemic external solution. However pretreatment of myocytes with AM630 (100 nM), not AM251 (500 nM), for 15 min abolished the inhibitory effect of anandamide (100 nM) on I_NCX_ in the simulated ischemic external solution (Figure. 4A and 4B). In addition, the effect of CB2 receptor agonist JWH133 on I_NCX_ in ventricular myocytes during simulated ischemia was also tested. JWH133 (100 nM) significantly inhibited the increase in I_NCX_ during simulated ischemia, and the effect of JWH133 was completely abolished by AM630 (Figure. 4C).The concentration of JWH133 was chosen according to previous report [24].

### Pretreatment of Gi/o protein specific inhibitor PTX inhibited the effects of anandamdie and JWH133 on I_NCX_ in the simulated ischemic external solution

**Figure 5:**
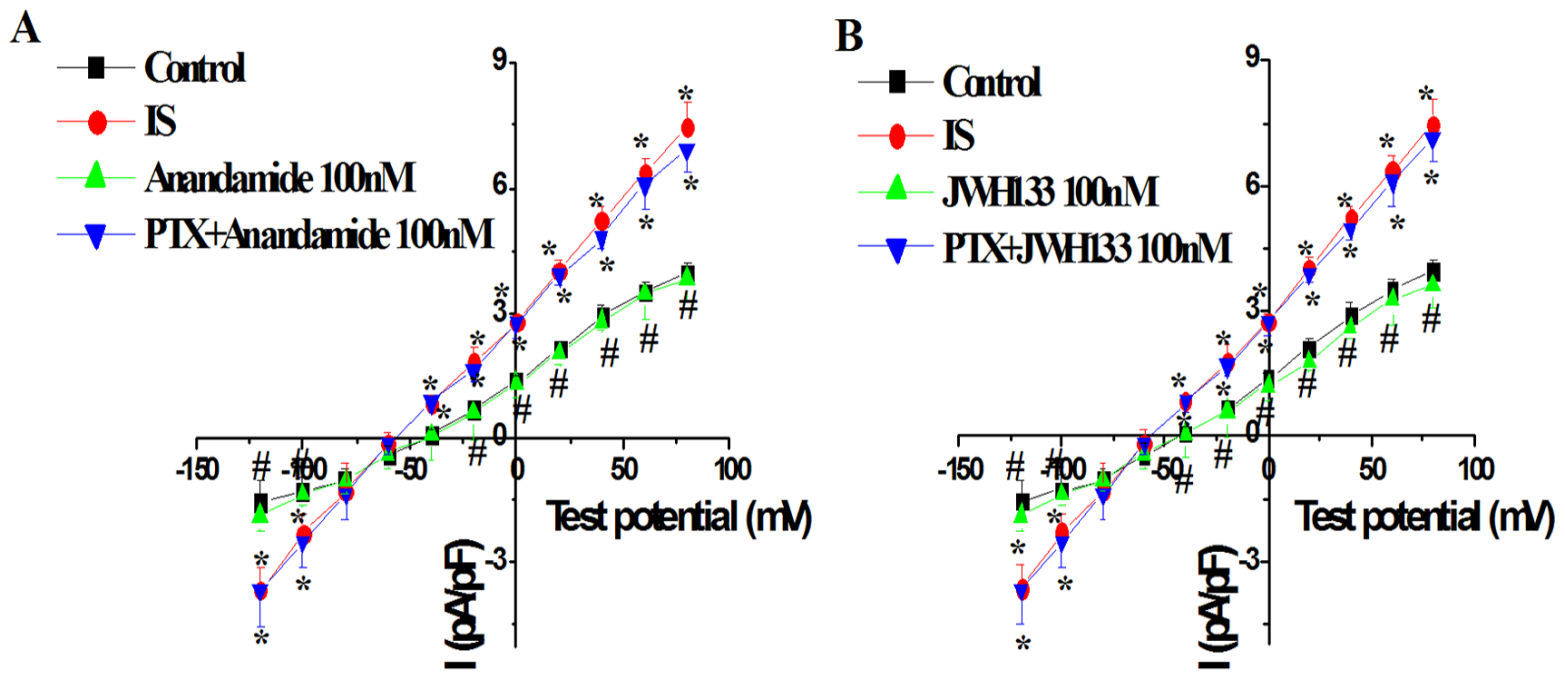
Effects of Gi/o protein antagonist pertussis toxin (PTX, 500 ng/mL) on anandamide-induced I_NCX_ change (A) and JWH133 -induced I_NCX_ change (B) in isolated rat ventricular myocytes in the simulated ischemic external solution. * *P*<0.05 vs Control, **^#^** *P*<0.05 vs IS, n = 10 in each group.

To evaluate the role of Gi/o proteins in transducing the anandamide-mediated I_NCX_ change, cells were pretreated with 500 ng/ml of PTX overnight [25]. The effects of anandamide and JWH133 on I_NCX_ in ventricular myocytes during simulated ischemia were tested after pretreatment with PTX overnight. PTX completely abolished inhibitory effects of anandamide (100 nM) and JWH133(100 nM) on I_NCX_ in the simulated ischemic external solution (Figure. 5).

### Effect of anandamide on [Ca^2+^]_i_ in ventricular myocyte

**Figure 6:**
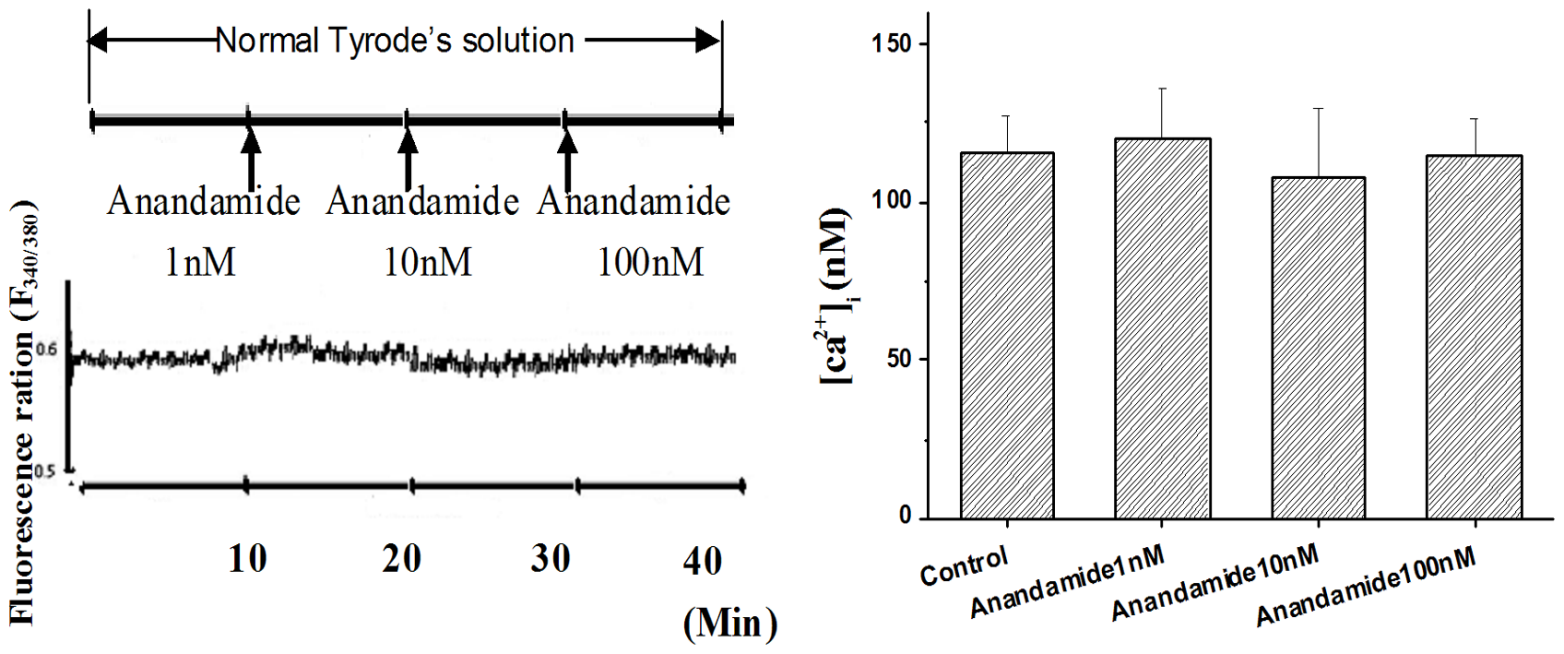
Effects of anandamide on [Ca^2+^]_i_ in isolated rat ventricular myocytes in normal Tyrode's solution. (A) Transient fluorescence ratios (340∶380) were observed in the single Fura-2-loaded ventricular myocyte in normal Tyrode's solution followed by exposure to anandamide 1 nM, 10 nM and 100 nM. (B) Summary data showing the anandamide (1 nM, 10 nM and 100 nM) had no effect on [Ca^2+^]_i_ in normal Tyrode's solution, n = 10 in each group.

**Figure 7:**
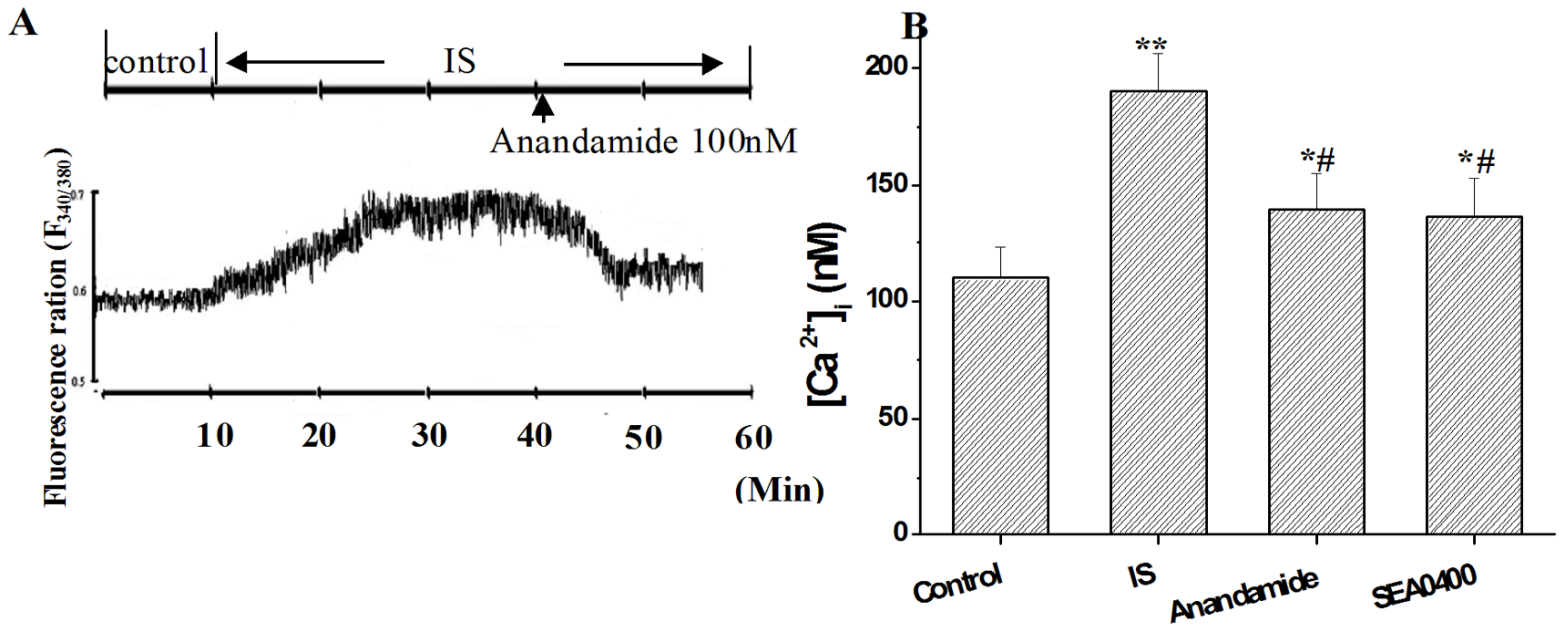
Effects of anandamide 100 nM or NCX inhibitor SEA0400 1 µM on [Ca^2+^]_i_ in ventricular myocytes in simulated ischemic solution. (A) Transient fluorescence ratios (340∶380) were observed in the single Fura-2-loaded ventricular myocyte in simulated ischemic solution (IS) followed by exposure to anandamide 100 nM. (B) Summary data showing the anandamide and SEA0400 reduced [Ca^2+^]_i_ partially in simulated ischemic solution. *P<0.05 **P<0.01 vs Control, ^#^ *P*<0.05 vs IS, n = 10 in each group.

The myocytes were perfused with normal Tyrode's solution, the [Ca^2+^]_i_ was recorded before and after bath application of anandamide at time points of 1, 5, 10, 15, 20, 25 and 30 min. The [Ca^2+^]_i_ had no change with application of anandamide at each time points (Figure. 6). After being perfused in normal Tyrode's solution for 10 min, the myocytes was perfused by simulated ischemic solution (containing 20 µM glibenclamide) for 30 min. Compared with the [Ca^2+^]_i_ in normal Tyrode's solution, simulated ischemic solution increased [Ca^2+^]_i_ significantly at 30 min (from 110.6 ± 13.2 nM to 190.8 ± 15.5 nM). Application of anandamide (100 nM) to the simulated ischemic solution for 15 min significantly inhibited [Ca^2+^]_i_ to 139.9 ± 15.8 nM, but the [Ca^2+^]_i_ was still higher than that in normal Tyrode's solution (Figure. 7B). Anandamide was added to the simulated ischemic solution 3 min before recording, the maximal effects of anandamide can be measured within 8 min of initial exposure. We also tested the effect of NCX inhibitor SEA0400 (1 µM) [26] on the [Ca^2+^]_i_ in simulated ischemic solution. SEA0400 also partially inhibited the increase in [Ca^2+^]_i_ in simulated ischemic solution (Figure. 7B).

### Role of CB1 and CB2 receptors in the effect of anandamide on [Ca^2+^]_i_ in simulated ischemic solution

**Figure 8:**
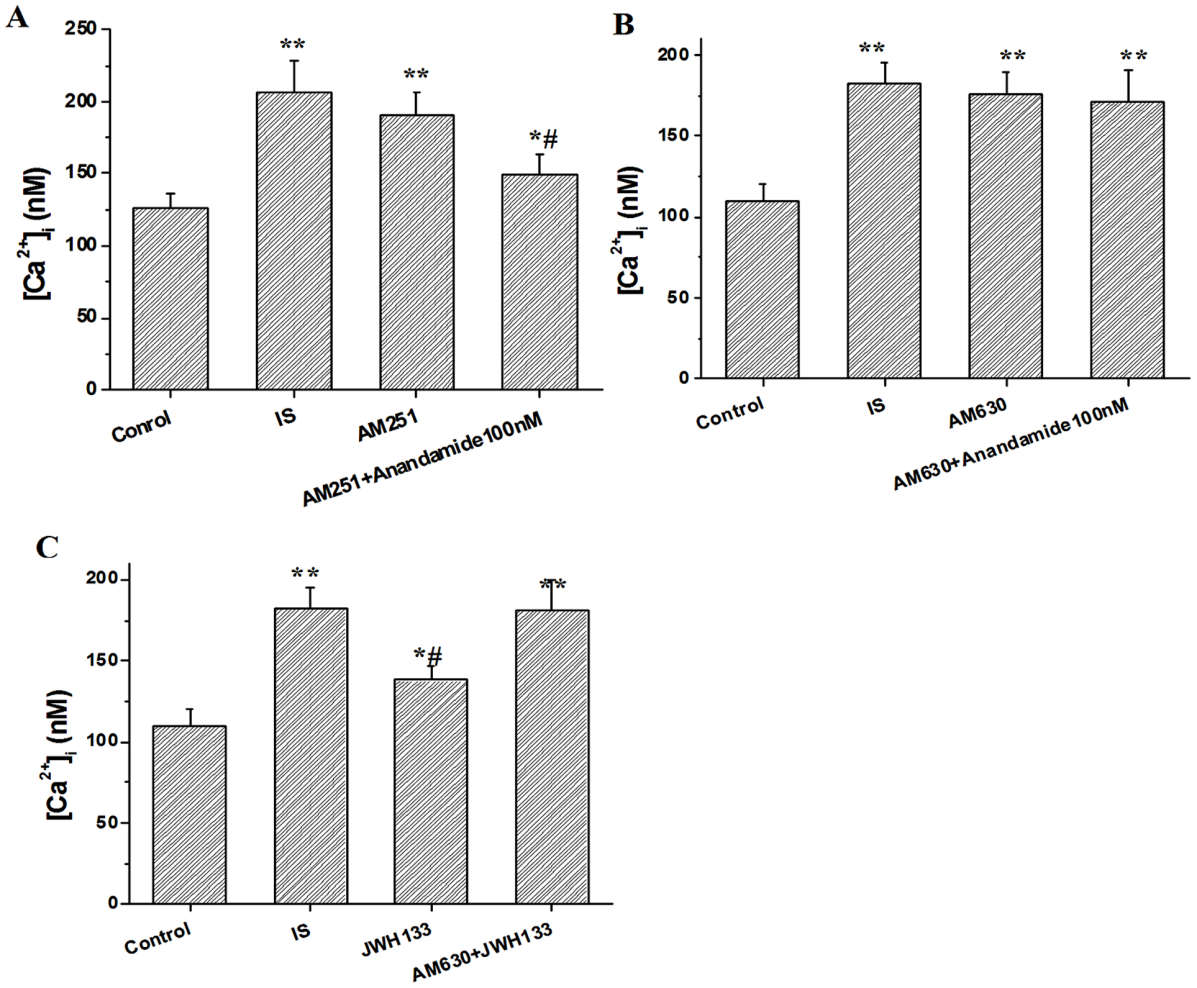
Effects of AM251 (500 nM) (A) and AM630 (100 nM) (B) on anandamide-induced [Ca^2+^]_i_ change, and CB2 receptor agonist JWH133 (100 nM) on [Ca^2+^]_i_ (C) in isolated rat ventricular myocytes in the simulated ischemic solution. *P<0.05, **P<0.01 vs Control, ^#^ *P*<0.05 vs IS, n = 10 in each group.

To determine the receptor subtypes that mediated the effect of anandamide on [Ca^2+^]_i_, The myocytes was perfused by normal Tyrode solution for 10 min before exposed to the simulated ischemic solution. Then, the effect of 100 nM anandamide on [Ca^2+^]_i_ in ventricular myocytes was tested after pretreatment of the myocytes with CB1 antagonist AM251 (500 nM) or CB2 antagonist AM630 (100 nM) for 15 min in the simulated ischemic solution. Blockade of CB1 receptors with AM251 (500 nM) or CB2 receptors with AM630 (100 nM) had no effect on basal [Ca^2+^]_i_ level in cardiac myocytes. However, pretreatment of cells with AM630 (100 nM), but not AM251 (500 nM), for 15 min abolished the inhibitory effect of anandamide (100 nM) on [Ca^2+^]_i_ in the simulated ischemic solution (Figure. 8A and 8B). In addition, CB2 receptor agonist JWH133 (100 nM) also partially inhibited the increase in [Ca^2+^]_i_ in simulated ischemic solution, and the effect was abolished by AM630 completely (Figure. 8C).

## Discussion

In this experiment, we studied the effect of anandamide on I_NCX_ and [Ca^2+^]_i_ when ventricular myocytes exposed to simulated ischemic solution, which mimics cardiac ischemia. The internal enviroment changes to hypoxia, acidosis, lactate, hyperkalaemia, glucose-free during ischemia, at present most of in vitro experiments use simulated ischemia solution to mimics ischemic conditions [21]. So we also use the simulated ischemic solution to mimics ischemic conditions according to previous study [19]–[21]. We found that anandamide had no effect on I_NCX_ and [Ca^2+^]_i_ in normal condition. However, both I_NCX_ and [Ca^2+^]_i_ were enhanced in simulated ischemic solution. Anandamide inhibited the enhancement of I_NCX_ and partially suppressed the increase in [Ca^2+^]_i_ in cardiac myocytes in simulated ischemic solution. These effects were mediated by CB2 but not CB1 receptors, and PTX sensitive Gi/o protein may be involved. These findings suggest that anandamide suppresses calcium overload through inhibition of I_NCX_ during ischemia.

Although anandamide and other cannabinoids have little effect on cardiovascular hemodynamics in conscious animals [27]–[29], it has been shown that anandamide decreases cardiac contractility in human atrial muscle and rat isolated hearts [30], [31]. [Ca^2+^]_i_ plays a crucial role in determining cardiac contraction and relaxation. However, we found that anandamide had little effect on I_NCX_ and [Ca^2+^]_i_ in cardiac myocytes in normal pH solutions. It seems that our findings are contradictory to the negative inotropic effects of anandamide. Previous studies have shown that cannabinoid decreases contractility of the heart and is associated with an increase in nitric oxide (NO) and cyclic guanine mono-phosphate (cGMP) production in the cardiac myocardium [32]. It has been shown that NO exerts its negative inotropic effect through a reduction of myofilament Ca^2+^ sensitivity mediated exclusively by a cGMP-PKG pathway without significantly changing the amplitude or kinetics of [Ca^2+^]_i_ [33]. Therefore, it is possible that the negative inotropic effects of anandamide is attributed to a reduction of myofilament Ca^2+^ responsiveness without alteration of [Ca^2+^]_i_.

We found that I_NCX_ (both reverse mode and forward mode) was enhanced obviously in simulated ischemic external solution at 30 min. Furthermore, the reversal potential was shifted to the negative potentials. The intracellular Na^+^ increases during ischemia through enhanced activity of Na^+^-H^+^ exchange (NHE) [34] or noninactivating Na^+^ channels [35]. It has been shown that persistent Na^+^ current and Na^+^/H^+^ exchanger activity contribute to the augmentation of the reverse NCX activity during hypoxia or acute ischemia in ventricular myocytes [36]. This rise in intracellular Na^+^ concentration, coupled with the depolarized plasma membrane, results in an increase in the reversal NCX activity to bring more Ca^2+^ into the cardiac myocytes [18]. Because NHE activity affects NCX, we inhibited NHE with cariporide in our study to merely determine the effect of anandamide on I_NCX_. In the simulated ischemic external solution, anandamide reduced both outward and inward I_NCX_, the reversal potential was also recovered to the level of normal external solution. It was reported anandamide had little effect on Na^+^ channel in cardiac myocytes [11], it is likely that anandamide inhibited I_NCX_ directly. However, there were some reports that I_NCX_ was inhibited during cardiac ischemia [19], [26]. In these studies, the ischemia time is less than 10 minutes, which is relatively short to observe the stable alteration of NCX activity. We measured the I_NCX_ after perfusion of simulated ischemic solution for 30 min. Therefore, this discrepancy is possibly due to the measurement in different time points after ischemia.

Increasing lines of studies indicate that a rise in [Ca^2+^]_i_ of myocardium during ischemia produces irreversible tissue damage. Therefore, a manipulation that reduces the rise in [Ca^2+^]_i_ can attenuate or delay the onset of irreversible cardiac injury [37]. Many mechanisms are involved in the increase in [Ca^2+^]_i_ during ischemia. In this regard, the rise in cytosolic Ca^2+^ during ischemia is due to Ca^2+^ entry through an increased activity of NCX in reverse mode [18]. Thus, inhibition of NCX has been shown to protectively reduced [Ca^2+^]_i_ [34], [38]. Furthermore, hearts from mutant mice lacking the NHE gene has been shown to reduce ischemia-reperfusion injury [18], [39]. We found that anandamide inhibited I_NCX_ in the simulated ischemic external solution. Therefore, we reasoned that anandamide decrease [Ca^2+^]_i_ through inhibition of I_NCX_ to protect cardiomyocyte against ischemia injury. Consistent with previous study [34], we found that the calculated [Ca^2+^]_i_ was increased during perfusion of simulated ischemic solution. Anandamide partially, but significantly, inhibited the increase in [Ca^2+^]_i_ in simulated ischemic solution. These result is consistent with the finding that anandamide elicited a concentration-dependent inhibition of depolarization-evoked Ca^2+^ transients in oligodendroglial somata [40],inhibited Ca^2+^ transients [41],and negatively modulated IP_3_-mediated nuclear Ca^2+^ release in isolated cardiac nuclei [42]. We used SEA0400, a selective inhibitor for NCX (both mode). It has been shown that SEA0400 inhibits 80% I_NCX_ at a concentration of 1 µM [38]. SEA0400 protects mouse cardiac myocytes from Ca^2+^ overload during I/R injuries [26] and reduce the ischemic-induced Ca^2+^ overload [38]. We found that NCX inhibitor SEA0400 also partially inhibited the increase in [Ca^2+^]_i_ in simulated ischemic solution. These data suggest that anandamide inhibits [Ca^2+^]_i_ during ischemia through inhibition of I_NCX_.

Two types of cannabinoid receptors, the CB1 and CB2 have been cloned [43], [44] and are widely expressed in the cardiovascular system such as blood vessels and cardiac tissue [5]. Both receptor types belong to a group of seven transmembrane-spanning receptors and are coupled to Gi/o-proteins and their activation leads to inhibition of adenylyl cyclase [4]. Anandamide is a natural constituent of the plasma membrane and considered to be CB1 and CB2 endogenous agonist because it exhibits pharmacological activities comparable to cannabinoids [45]. In this study, we found that blockade of CB2 receptors with selective antagonist AM630 completely blocked the inhibitory effect of anandamide on both I_NCX_ and [Ca^2+^]_i_ in cardiac myocytes in simulated ischemic solution. To further clarify the effect of CB2 receptor, the effect of CB2 specific agonist JWH133 was tested. The effects of JWH133 was similar to anandamide, which also inhibited the enhancement of I_NCX_ and partially suppressed the increase in [Ca^2+^]_i_ in cardiac myocytes in simulated ischemic solution. However, a CB1 receptor antagonist AM251 failed to affect the inhibitory effects of anandamide on I_NCX_ and [Ca^2+^]_i_, even at higher concentrations. These findings indicated that the inhibitory effects of anandamide on I_NCX_ and [Ca^2+^]_i_ were mediated by CB2 receptor but not CB1 receptor. Many studies have emphasized the role of CB2 receptors in cardioprotection. For example, it has been shown that blockade of CB2 receptors eliminates cardiac protective effect of endocannabinoids in rat isolated hearts exposed to low-flow ischemia and reperfusion [46], [47]. Furthermore, a recent study has shown that a single dose of the CB2 agonist JWH-133 reduced infarct size during cardiac infarction [48].

Recent studies have reported that NCX function is regulated by tyrosine phosphorylation [49]. CB2 receptor is coupled to Gi/o, and phosphorylation status of NCX may be affected. In this study,PTX was used to test whether Gi/o protein is involved in the effect of CB2 receptor. PTX completely inhibited not only the effect of anandamide on I_NCX_, but also the effect of CB2 receptor agonist JWH133 on I_NCX_. Thus, at least we can deduce that anandamide inhibites I_NCX_ through CB2 receptor via PTX sensitive Gi/o proteins. But the next signaling pathway after activation of Gi/o need further exploration.

In conclusion, these findings suggest that anandamide suppresses calcium overload through inhibition of I_NCX_ during perfusion with simulated ischemic solution, the effects may be mediated by CB2 receptor via PTX-sensitive Gi/o proteins.
